# [Corrigendum] Decreased RNA‑binding protein IGF2BP2 downregulates NT5DC2, which suppresses cell proliferation, and induces cell cycle arrest and apoptosis in diffuse large B‑cell lymphoma cells by regulating the p53 signaling pathway

**DOI:** 10.3892/mmr.2023.12954

**Published:** 2023-02-08

**Authors:** Yuying Cui, Yu Wen, Chao Lv, Dongmei Zhao, Yu Yang, Hongbin Qiu, Chennan Wang

Mol Med Rep 26: 286, 2022; DOI: 10.3892/mmr.2022.12802

Subsequently to the publication of this paper, the authors have realized that they included the incorrect data in Fig. 5 for the p21 blots on p. 8. The corrected version of Fig. 5, featuring the data that were intended to have been included in this figure for the p21 blots, is shown on the next page.

Furthermore, the authors wish to make the following changes to the text in the paper (changes are highlighted in bold):

i) on p. 2, left-hand column, line 16, the final sentence in the penultimate paragraph of the Introduction should have read as: “However, the functions of **RNA-binding protein (RBP)** IGF2BP2, and the interactions between IGF2BP2 and NT5DC2 in DLBCL remain to be explored.”;

ii) In the Materials and methods section, subsection “*RNA pull-down assay*”, the first sentence should be revised to: “An RNA pull down kit (cat. no. P0202: Geneseed) **was** used to perform RNA pull down assay according to the manufacturer's instructions.”;

iii) In the Results section on p. 3, the subsection “*NT5DC2 is upregulated in DLBC cells and knockdown of NT5DC2 inhibits proliferation of DLBC cells.*”, the second sentence should be revised as follows: “mRNA and protein expression of NT5DC2 was highest in OCI-Ly7 cells compared with **that** in the other DLBC cell lines (Fig. 1A).”;

iv) The first two sentences in the following paragraph should have read as follows: “mRNA and protein expression levels of NT5DC2 were decreased in DLBC cells transfected with shRNA-NT5DC2#1/2 compared with **the untransfected group**, and were lower in the shRNA-NT5DC2#2 group compared with **the shRNA-NT5DC2#1 group** (Fig. 1B).”; and v) On p. 7, left-hand column, the sentence commencing on line 59 should have read as: “The present results indicated that NT5DC2 was increased in DLBCL cells...”.

Note that these errors did not significantly affect either the results or the conclusions reported in this paper, and all the authors agree with the publication of this corrigendum. Furthermore, the authors thank the Editor of *Molecular Medicine Reports* for allowing them the opportunity to publish this corrigendum, and apologize to the readership for any inconvenience caused.

## Figures and Tables

**Figure 5.**
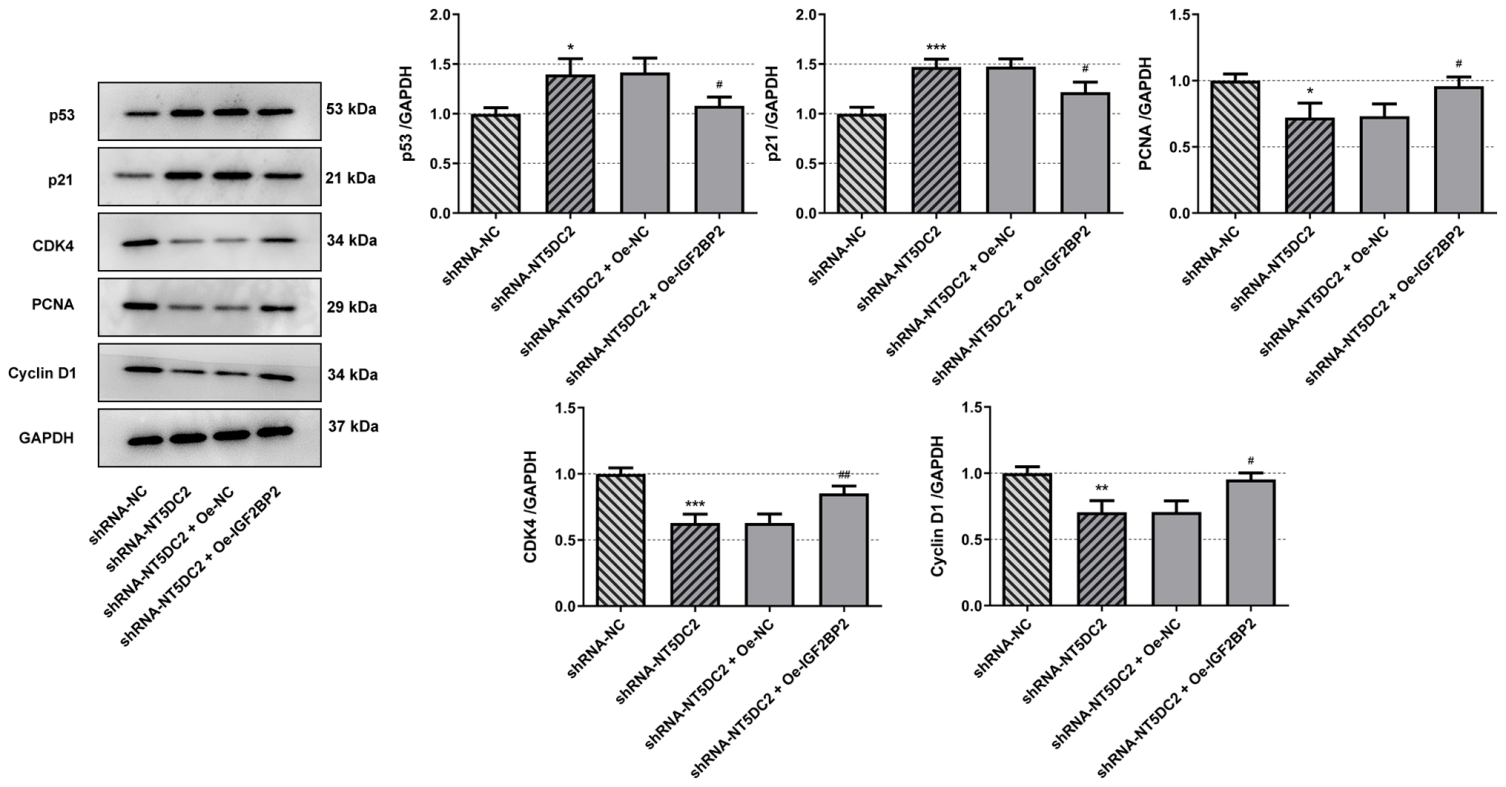
NT5DC2 regulates the p53 signaling pathway. The expression levels of p53 signaling pathway-associated proteins were analyzed by western blotting. *P<0.05, **P<0.01 and ***P<0.001 vs. shRNA-NC; ^#^P<0.05 and ^##^P<0.01 vs. shRNA-NT5DC2 + Oe-NC. NT5DC2, 5’-nucleotidase domain-containing 2; shRNA, short hairpin RNA; NC, negative control; oe, overexpression.

